# *Bacillus toyonensis* SAU-19 Ameliorates Hepatic Insulin Resistance in High-Fat Diet/Streptozocin-Induced Diabetic Mice

**DOI:** 10.3390/nu13124512

**Published:** 2021-12-17

**Authors:** Samuel Kumi Okyere, Lei Xie, Juan Wen, Yinan Ran, Zhihua Ren, Junliang Deng, Yanchun Hu

**Affiliations:** 1 Key Laboratory of Animal Diseases and Environmental Hazards of Sichuan Province, College of Veterinary Medicine, Sichuan Agricultural University, Chengdu 611130, China; samuel20okyere@gmail.com (S.K.O.); wsxielei@gmail.com (L.X.); juanwen881010@163.com (J.W.); ranyinan17@163.com (Y.R.); zhihua_ren@126.com (Z.R.); dengjl213@126.com (J.D.); 2 New Ruipeng Pet Healthcare Group Co., Ltd., Shenzhen 518000, China

**Keywords:** type 2 diabetes, hepatic insulin resistance, *Bacillus toyonensis* SAU-19, glucose synthesis

## Abstract

Insulin resistance (IR) is a hallmark of type 2 diabetes mellitus (T2DM). This study was performed to investigate the antidiabetic effect of *Bacillus toyonensis* SAU-19 and its possible mechanisms of action in mice with type 2 diabetes mellitus (T2DM). Thirty SPFKM mice were randomly assigned to three groups: control, diabetic model, and diabetes + *Bacillus toyonensis* SAU-19 group. After 35 days, blood was collected for biochemical analysis and liver tissue samples for histopathological analysis using H&E staining, qPCR, and ELISA. The results showed that the administration of *B. toyonensis* SAU-19 significantly improved the blood glucose, hepatic insulin resistance, and morphological changes of the liver characterized by significant improvement of dyslipidemia, glycogen synthesis, and antioxidant status (*p* < 0.05), indicating the strains’ ameliorating effects on hepatic insulin resistance in T2DM. In conclusion, the probiotic strain *(B. toyonensis* SAU-19) inhibits T2DM by reducing insulin resistance, improving antioxidant status, and downregulating genes related to glucose synthesis; hence, it may be used in treating diabetes and other metabolic disorders. This study provides the basis for further studies into the molecular mechanisms of *B. toyonensis* SAU-19 in treating T2DM.

## 1. Introduction

Type 2 diabetes mellitus (T2DM) is a metabolic syndrome, chiefly associated with chronic hyperglycemia as a result of insulin resistance and inadequate insulin secretion [1]. T2DM is one of the major diseases that affect human health and life globally [2]. In the year 2014, it was estimated that about 422 million adults were suffering from diabetes [3]. Furthermore, in 2010, the. global frequency of adult diabetes was estimated as 285 million and was speculated to increase to about 439 million by 2030 [4]. The liver is the key organ involved in the preservation of glucose homeostasis as it controls the balance between gluconeogenesis and glycogen synthesis in the body [5]. Insulin resistance elevates gluconeogenesis and decrease glycogen synthesis in the liver, resulting in hyperglycemia [6]. The liver stores excessive lipids as fat droplets and the accumulation of these fat droplets may cause inflammation, insulin resistance, and diabetes [7]. Numerous studies have reported on the use of probiotics in treating diabetes and other metabolic diseases. In addition, since the effect and mechanisms of probiotics are species specific, there is a need to investigate specific probiotic strains and elucidate their efficiency for managing T2DM.

*Bacillus toyonensis*, a bacteria strain from the *Bacillus cereus* family, has been proven safe and is being used as a probiotic for many animals, such rabbits, pigs, chickens, and cattle [8,9]. Numerous studies have revealed the probiotic activities of this bacteria [10]. *Bacillus toyonensis* has been reported to improve feed conversion ratios and reduce post-weaning diarrhea and mortality in piglets [11] as well as reducing the activities of pathogenic bacteria in the gut [12]. However, studies on the probiotic bacteria’s antidiabetic properties have not been reported in the literature.

Therefore, in this study, we investigated the effect of the probiotic *Bacillus toyonensis* strain SAU-19, which was identified in our lab, on T2DM in mice. In our previous study, the *Bacillus toyonensis* strain SAU-19 isolated from *Ageratina adenophora* plant [13,14] showed tolerance to simulated gastrointestinal tract conditions, and improved the growth performance, antioxidant capacity, anti-inflammatory effects, and gut integrity in our animal experiment (unpublished), which provided a basis for the beneficial effects of *Bacillus toyonensis* SAU-19 in vivo. This study provides a theoretical basis for future use of the *B. toyonensis* strain SAU-19 in treating diabetes and other metabolic diseases.

## 2. Material and Methods

### 2.1. Sample Collection

Culture media were purchased from Qingdao Hope Bio-Technology Co., Ltd., Qingdao, China and streptozotocin (STZ) was purchased from Solarbio solabao Beijing solabao Technology Co., Ltd., China. Mice, basal, and high-fat diet (Table 1) were purchased from the Chengdu Dashuo Experiment Animal Co., Ltd., Chengdu, China. *Bacillus toyonensis* SAU-19 (Accessory number: MW287198; Collection Preservation number: CCTCC NO: M 20211138) was obtained from the College of Veterinary Medicine (Professor Yanchun Hu’s lab), Sichuan Agricultural University, China.

### 2.2. Preparation of Probiotic Bacteria Suspensions

*B. toyonensis* SAU-19 was transferred twice in LB broth and incubated anaerobically at 37 °C for 72 h. The bacterial cells were collected by centrifugation (3500× *g*, 5 min), and washed twice in 0.85% NaCl (Sigma), and then resuspended in 0.85% NaCl to a final concentration of 10^6^ CFU/mL, and stored at 4 °C.

### 2.3. Experimental Animal and Design

Thirty male specific-pathogen-free Kun min mice (SPFKM) (5 weeks old; BW 25–30 g) were purchased from the Chengdu Dashuo Experiment Animal Co., Ltd., Chengdu, China. The animals were housed in an experimental animal house at Sichuan Agricultural University at a constant temperature (22 ± 2 °C) and humidity (65 ± 5%) under a 12-h light/12-h dark cycle with free access to food and water. This study was approved by the Institutional Animal Care and Use Committee of Sichuan Agricultural University, Sichuan, China, under the permit number DKY-B2019603005.

The mice diabetes model was established by the administration of a high-fat diet (HFD) for 6 weeks and intraperitoneal injection of streptozotocin (STZ) solution (dissolved in a 0.01 M citrate buffer, pH 4.5 (Solarbio Science and Technology Co., Ltd., Beijing, China) at a dose of 35 mg/kg body weight for 3 consecutive days [15]. Then, 72 h after the injection, fasting blood glucose (FBG) was measured using a blood glucose meter (Bayer). The diabetes model was identified as successfully prepared in cases where random blood glucose level > 11.1 mmoL/L [1]. Mice injected with equivalent amounts of with equivalent amounts of precooling citrate buffer solution, pH 4.5 were used as controls (*n* = 10). The model mice were randomly divided into a diabetic group (DG) (*n* = 10) that was fed HFD + 1 mL 0.9% normal saline daily in drinking water and a diabetic + *B. toyo* SAU-19 group (DG + *B. toyo* SAU-19) (*n* = 10) that was fed HFD + 1 mL of 1 × 10^6^ CFU mL^−1^ *B. toyo* SAU-19 in drinking water as well as a control group (C) (*n* = 10) fed a basal diet and 1 ml of 0.9% normal saline for 35 days. Feed and water intake was monitored and recorded daily throughout the experimental period. Feed and clean water were provided ad libitum. Water bottles were washed every week and fresh drinking water was placed in it for the next week’s administration. The bedding material (wood shavings) was also changed weekly. To administer the *B. toyo* SAU-19, new stocks were generated each week in LB, and their viability was monitored by serial dilution and viable cell count using LB agar, respectively.

### 2.4. Oral Glucose Tolerance Test

An oral glucose tolerance test (OGTT) was performed in the last week of *B. toyo* SAU-19 administration. Mice were fasted for 12 h and blood glucose was determined (time = 0 min). Then, mice were orally administered glucose (2 g kg^−1^ BW) and blood glucose levels were measured at 30, 60, 90, and 120 min.

### 2.5. Blood and Tissue Sample Collection

At the end of the experiment (week 12), mice were fasted for 12 h and anesthetized with sevoflurane. Blood samples were collected from the inferior vena cava and centrifuged at 4000× *g* for 10 min at 4 °C, and then the serum was collected and stored at −80 °C for further assays. Liver tissue samples were quickly removed, rinsed, and stored at −80 °C or fixed in 10% paraformaldehyde solution.

### 2.6. Biochemical Parameters

Liver glycogen and serum insulin were determined using ELISA kits (Jiangsu Jingmei biological Technology company limited, Jiangsu, China). Lipid profiles, including total cholesterol (TC), total triglyceride (TG), LDL-cholesterol (LDL-C), and HDL cholesterol (HDL-C), were determined by commercial kits (Jiangsu Jingmei biological Technology company limited, Jiangsu, China). Homeostatic model assessment of insulin resistance (HOMAIR), used to quantify insulin resistance, was calculated as: HOMA-IR = Fasting blood glucose (mmol L^−1^) × Fasting blood insulin (mU L^−1^)/22.5 [16]. The levels of glutathione (GSH), malondialdehyde (MDA), and superoxide dismutase (SOD) in mice serum and livers were also measured using commercial kits from Jiangsu Jingmei biological Technology company limited, Jiangsu, China.

### 2.7. Liver Histological Analysis

Livers fixed in 4% paraformaldehyde were embedded in paraffin and sectioned for 5 μm thick. Hematoxylinensin (H&E) staining was used for liver pathological evaluation. H&E staining kits were purchased from Jiancheng Bioengineering Institute (Nanjing, China). All kits were used according to the corresponding manufacturers’ instructions. Liver injury was numerically recorded following the method of Chen et al. [17].

### 2.8. Enzyme-Linked Immunosorbent Assay

Parts of the liver tissues were washed with PBS. Then, 0.1 g of the sample tissue was weighed and homogenized with 0.9 mL of ice-cold PBS in a glass homogenizer, and then the mixture was centrifuged (3000 rpm, 20 min) to obtain the supernatant. Furthermore, we determined the protein concentration in the supernatant using a Total Protein Assay kit (Nanjing Jiancheng Bioengineering Institute, Nanjing, China). The supernatants were used to determine the concentrations of IL-1β, TNF-α, IL-4, and IL-10 using a commercial mice ELISA kit (Jiangsu Jingmei Biological Technology Co., Ltd., Jiangsu, China), respectively. The level of sensitivity of each kit was 0.1 pg/mL for each cytokine [18].

### 2.9. Reverse Transcription-Quantitative Polymerase Chain Reaction (RT-qPCR)

Samples of liver tissues (30 mg/mouse) were snap-frozen with liquid N_2_ and then immediately ground into powder using a ceramic mortar. Total RNA from each sample was extracted using an Animal Total RNA Isolation Kit (Sagon Biotech, Shanghai, China) according to the manufacturer’s instructions. After confirming the isolated RNA concentration and purity using a NanoDrop One system (Thermo Fisher Scientific, Waltham, MA; OD260/280 ≈ 1.9–2.0), triplicate aliquots (each 1 µg) were removed, loaded into wells, and cDNA was prepared using a PrimeScrip RT reagent kit (Takara, Tokyo Japan). Thereafter, qRT-PCR was performed using a SYBR Premix ExTaq (Takara) and a CFX96 thermal cycler (BioRad, Hercules, CA, USA). The PCR conditions were as follows: 95 °C for 5 min, followed by 40 cycles of 95 °C, 15 s for denaturation, 60 °C, 60 s for annealing at and 70 °C, 25 s for extension. Each qRT-PCR reaction was performed with volumes of 10 µL containing 5 µL of TB Green TM Premix (Takara), 1 µL of forward and reverse primers, 1 µL of cDNA, and 2 µL of DNase/RNase-Free Deionized Water (Tiangen, Beijing, China). The primers used to analyze the genes of interest were designed from NCBI genBank and are shown in Table 2. The relative gene expression in each sample was normalized to an internal control (β-actin); data analysis was performed using the 2^−ΔΔCt^ method. All samples were evaluated in triplicate.

### 2.10. Statistical Analysis

Statistical analysis of the data collected (from various independent experiments) was performed using GraphPad Prism 5.04 software (GraphPad Software, Inc., La Jolla, CA, USA) and SPSS 20 Statistical Analysis Software (SPSS Inc., Chicago, IL, USA). The Shapiro–Wilk Test was used to test the normality of the data. All experimental results are presented as mean ± SD, and statistical significance were determined by one-way analysis of variance (ANOVA) followed by the Tukey’s test. The values were significantly different at *p* < 0.05.

## 3. Results

### 3.1. Effects of B. toyonensis Strain SAU-19 on Growth Performance in HFD/STZ-Induced T2DM Mice

**Figure 1:**
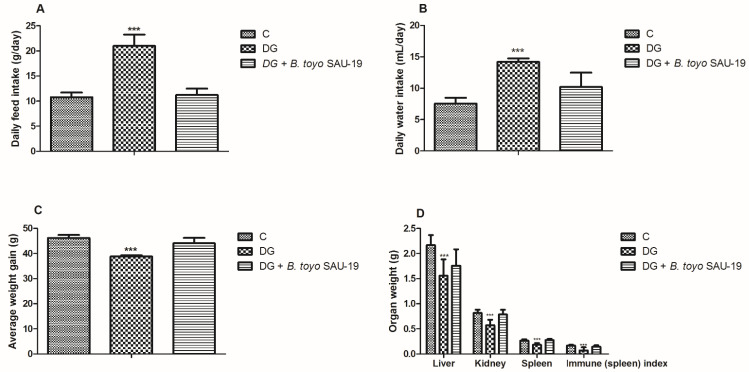
Effects of *Bacillus toyonensis* SAU-19 on the growth performance in HFD/STZ-induced T2DM mice. (**A**) Daily feed intake (g/day). (**B**) Daily water intake (mL/day). (**C**) Average weight gain (g). (**D**) Organ weight (g) and immune (spleen) index. Values are shown as mean ± Sd. Bars with *** are statistically different (*p* < 0.05), DG compared with the control (C) and *B. toyo* SAU-19 groups (*n* = 8).

From the results, during the experimental trial, we observed a significant increase in feed and water intake in the DG group compared to the control (C) and *B. toyo* SAU-19 groups (Figure 1A,B, *p* < 0.05), typical of T2DM. However, there was no difference between the control (C) and *B. toyo* SAU-19 groups in feed and water intake (*p* > 0.05). Furthermore, we also observed a decrease in the weight gain in the DG group after 35 days compared to the control (C) and *B. toyo* SAU-19 groups even though feed intake was high in the DG group (Figure 1C, *p* < 0.05). No difference existed between the control (C) and *B. toyo* SAU-19 groups. Moreover, we observed a significant decrease in liver, kidney, and spleen weights of the DG group compared to the control (C) and *B. toyo* SAU-19 groups (Figure 1D, *p* < 0.05). The immune index scores for the DG group were significantly lower than that of the control (C) and *B. toyo SAU*-19 groups (Figure 1D, *p* < 0.05). There was no difference in the organ weights and immune index scores of the control (C) and *B. toyo* SAU-19 groups (*p* > 0.05). The immune index was calculated as: Immune index = spleen weight (g)/Body weight (g).

### 3.2. Effects of B. toyonensis Strain SAU-19 on Blood Glucose Levels and Oral Glucose Tolerance Test (OGTT) in HFD/STZ-Induced T2DM Mice

**Figure 2:**
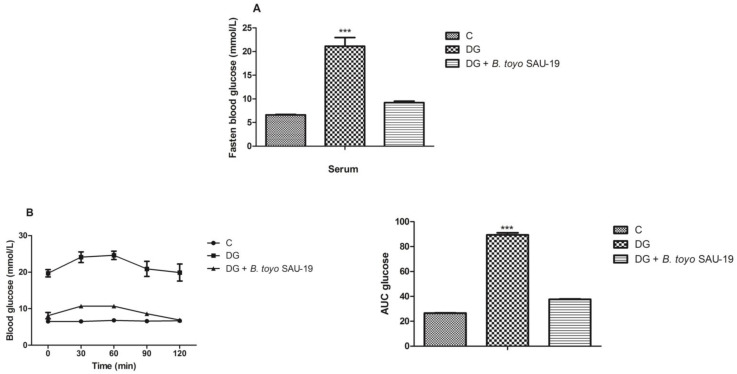
Effects of *Bacillus toyonensis* SAU-19 on blood glucose tolerance in HFD/STZ-induced T2DM mice. (**A**) Fasted blood glucose (mmol/L). (**B**) Oral glucose test (mmol/L). Values are shown as mean ± Sd. Bars with *** are statistically different (*p* < 0.05), DG compared with the control (C) and *B. toyo* SAU-19 groups (*n* = 6).

As shown in Figure 2A, the fasted blood glucose level of the DG group after the 35-day administration period was higher as compared to the control (C) and *B. toyo* SAU-19 groups (*p* < 0.05). However, there was no difference in the fasted blood glucose between the control (C) and *B. toyo* SAU-19 groups. Furthermore, the glucose area under the curve (AUC) for the OGTT value in DG mice was significantly larger than that of the control (C) (Figure 2B, *p* < 0.05). The glucose AUC was significantly lowered following oral administration of the *B. toyonensis* strain SAU-19 to mice compared to the DG group (*p* < 0.05), with no significant difference compared to the control (C) group (*p* > 0.05).

### 3.3. Effects of B. toyonensis Strain SAU-19 on Biochemical Parameters in HFD/STZ-Induced T2DM Mice

**Figure 3:**
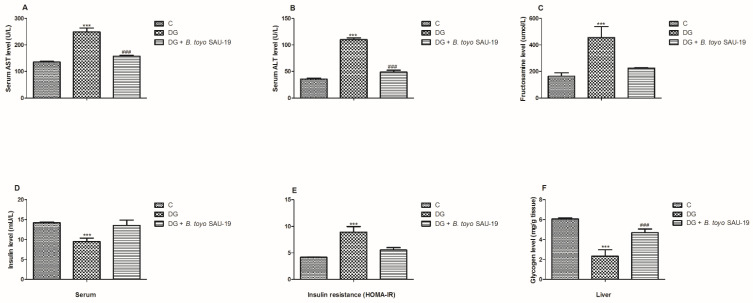
Effects of *Bacillus toyonensis* SAU-19 on biochemical parameters in HFD/STZ-induced T2DM mice. (**A**) Levels of AST in blood serum (U/L). (**B**) Levels of ALT in blood serum (U/L). (**C**) Levels of fructosamine in serum (μmol/L). (**D**) Insulin level in the blood serum (mU/L). (**E**) Insulin resistance indicator. (**F**) Glycogen content in liver (mg/g tissue). Values are shown as mean ± Sd. *** *p* < 0.05, DG compared with the control (C) and *B. toyo* SAU-19 groups. ^###^ *p* < 0.05, *B. toyo* SAU-19 compared with the control (C) group (*n* = 6). AST—Aspartate Aminotransferase, ALT—Alanine Aminotransferase.

As shown in Figure 3A–D, the levels of AST, ALT, fructosamine, and HOMA-IR in the diabetic (DG) group were significantly higher compared to the control (C) group (*p* < 0.05). However, administration of *B. toyonensis* SAU-19 significantly reduced the levels of AST, ALT, fructosamine, and HOMA-IR compared to the DG group mice (*p* < 0.05), but these parameters were not significantly different compared to the control (C) group.

The liver glycogen content and serum insulin levels were significantly lower in the DG group compared to the control (C) and *B. toyo* SAU-19 groups (Figure 3E, *p* > 0.05). In addition, the liver glycogen content was significantly lower in the *B. toyo* SAU-19 groups compared to the control (C) group (Figure 3E, *p* < 0.05). There was no difference in the insulin levels between the *B. toyo* SAU-19 groups and control (C) groups.

### 3.4. Effects of B. toyonensis Strain SAU-19 on Lipid Profiles in HFD/STZ-Induced T2DM Mice

**Figure 4:**
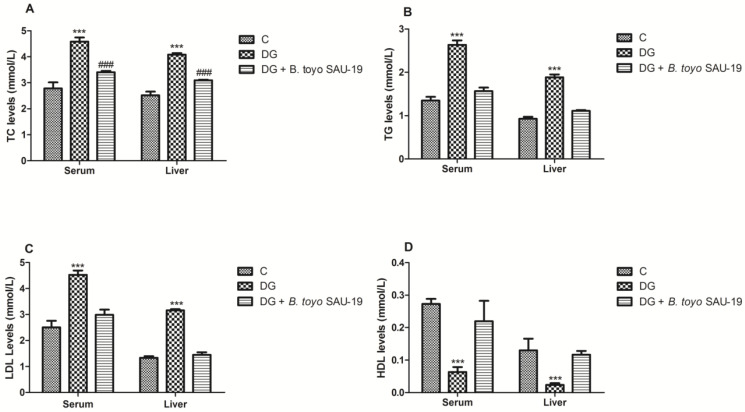
Effects of *Bacillus toyonensis* SAU-19 on lipid profiles in HFD/STZ-induced T2DM mice. (**A**) Levels of TC in blood and liver serum (mmol/L). (**B**) Levels of TG in blood and liver serum (mmol/L). (**C**) Levels of LDL-C in blood and liver serum (mmol/L). (**D**) Levels of HDL-C in blood and liver serum (mmol/L). Values are shown as mean ± Sd. *** *p* < 0.05, DG compared with the control (C) and *B. toyo* SAU-19 groups. **^###^** *p* < 0.05, *B. toyo* SAU-19 compared with the control (C) group (*n* = 6). TC—Total cholesterol, TG—Triglyceride, LDL-C—Low-density lipoprotein cholesterol, HDL-C—High-density lipoprotein cholesterol.

The effects of *B. toyonensis* strain SAU-19 on the lipid profile are shown in Figure 4. TC, TG, and LDL-C levels in the diabetic group (DG) were higher compared to the control (C) group in both the serum and liver (Figure 4A–C, *p* < 0.05). However, these parameters were significantly attenuated in the *B. toyo* SAU-19 group (*p* < 0.05). Moreover, the TC levels in the *B. toyo* SAU-19 group were higher as compared to the control (C) group (Figure 4A, *p* < 0.05). There were no significant differences in the TG and LDL-C levels between the *B. toyo* SAU-19 group and control (C) group. The HDL-C levels in the diabetic group (DG) were reduced as compared to the *B. toyo* SAU-19 group and control (C) group (Figure 4D, *p* < 0.05); however, there were no significant differences in the HDL-C levels between the *B. toyo* SAU-19 group and control (C) group.

### 3.5. Effects of B. toyonensis Strain SAU-19 on Antioxidant Activity in HFD/STZ-Induced T2DM Mice

**Figure 5:**
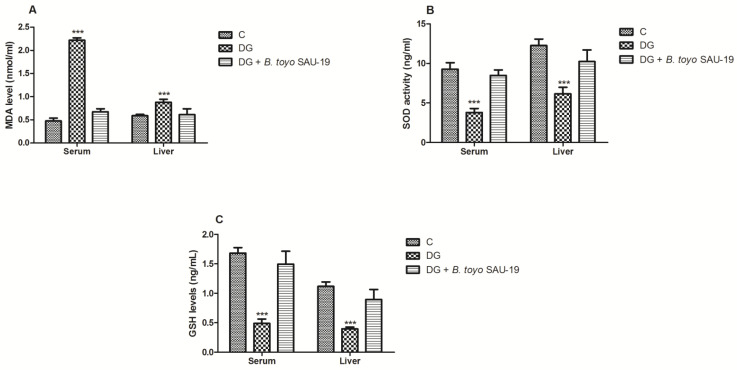
Effects of *Bacillus toyonensis* SAU-19 on antioxidant activity in HFD/STZ-induced T2DM mice. (**A**) Levels of MDA in blood and liver serum (nmol/L). (**B**) Levels of SOD in blood and liver serum (ng/mL). (**C**) Levels of GSH in blood and liver serum (ng/mL). Values are shown as mean ± Sd. Bars with *** are statistically different (*p* < 0.05), DG compared with the control (C) and *B. toyo* SAU-19 groups (*n* = 6). MDA—malondialdehyde, SOD—superoxide dismutase, GSH—glutathione.

The variations in the antioxidant status of the mice livers are presented in Figure 5. As compared to the control (C) group, the oxidative stress parameter (MDA) was increased in the DG whereas the antioxidative stress components (SOD and GSH) in the DG group were greatly decreased (Figure 5A–C, *p* < 0.05). However, the administration of the *B. toyonensis strain SAU-19* reverted these effects by increasing the levels of antioxidative stress components (SOD and GSH) and reducing the levels of the oxidative stress component MDA (*p* < 0.05). There was no significant difference in both the oxidative stress and antioxidant components between the *B. toyo* SAU-19 group and control (C) group (*p* > 0.05).

### 3.6. Effects of B. toyonensis Strain SAU-19 on Liver Histological Injury in HFD/STZ-Induced T2DM Mice

**Figure 6:**
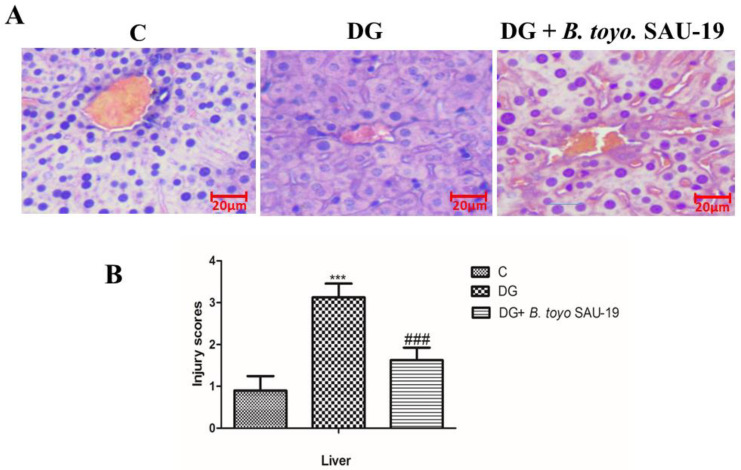
Effects of *Bacillus toyonensis* SAU-19 on histology (×200) in HFD/STZ-induced T2DM mice. (**A**) Photograph of histopathological staining in treatment groups (Scale = 20 µm). (**B**) Histological necrosis injury scores of the liver section of mice. Values are shown as mean ± Sd. *** *p* < 0.05, DG compared with the control (C) and *B. toyo* SAU-19 groups. ^###^ *p* < 0.05, *B. toyo* SAU-19 compared with the control (C) group C = Control, DG = Diabetic group, and DG + *B. toyo* SAU-19 (*n* = 5).

Histological analysis of the liver showed the well-ordered structure of the hepatic lobules in the control group’s liver sections, characterized by clear hepatic morphology and a centered nucleus. However, the diabetic mice group showed distortions in the hepatic lobule characterized by widespread degeneration, necrosis, and inflammation of the hepatocytes. These pathological disorders were noticeably improved by *B. toyonensis* SAU-19 feeding (Figure 6). Furthermore, SAU-19-fed mice had a significantly lower liver injury score compared to the HFD/STZ-fed mice; however, the injury scores of the SAU-19 group were significantly higher compared to the control group (Figure 6B, *p* < 0.05).

### 3.7. Effects of B. toyonensis Strain SAU-19 on Relative mRNA and Protein (ELISA) Expression of Genes Related to Inflammation in Liver Tissues of HFD/STZ-Induced T2DM Mice

**Figure 7:**
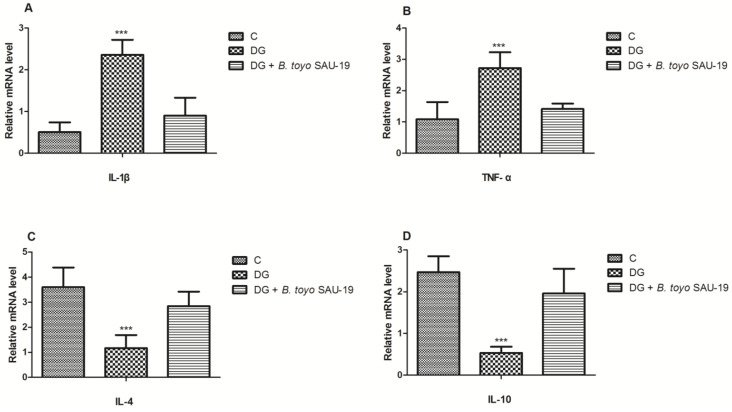
Effects of *Bacillus toyonensis* SAU-19 on relative mRNA expression of pro- and anti-inflammation-related cytokines in HFD/STZ-induced T2DM mice. (**A**,**B**) mRNA expression levels of pro-inflammation cytokines. (**C**,**D**) mRNA expression of anti-inflammatory cytokines. Values are shown as mean ± Sd. Bars with *** are statistically different (*p* < 0.05). DG compared with the control (C) and *B. toyo* SAU-19 groups (*n* = 6).

**Figure 8:**
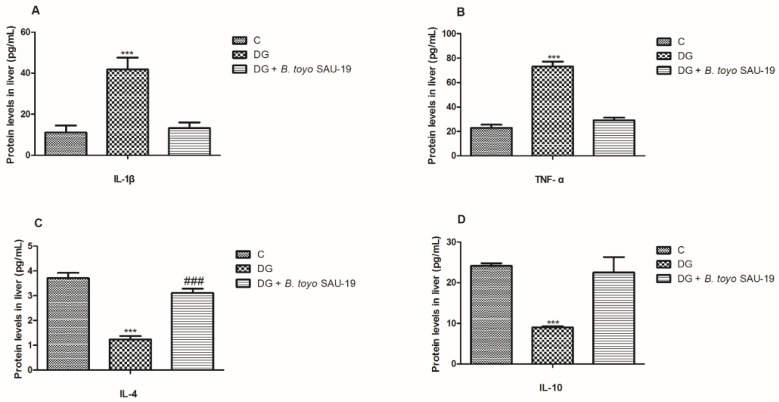
Effects of *Bacillus toyonensis* SAU-19 on relative protein expression (ELISA) of pro- and anti-inflammation-related cytokines in HFD/STZ-induced T2DM mice. (**A**,**B**) Protein (ELISA) expression levels of pro-inflammation cytokines. (**C**,**D**) Protein (ELISA) expression of anti-inflammatory cytokines. Values are shown as mean ± Sd. *** *p* < 0.05, DG compared with the control (C) and *B. toyo* SAU-19 groups. ^###^ *p* < 0.05, *B. toyo* SAU-19 compared with the control (C) group (*n* = 6).

As shown in Figure 7 and Figure 8, the mRNA and protein expression levels of proinflammatory cytokines (IL-1β and TNF-α) were significantly elevated whereas anti-inflammatory cytokines (IL-4 and IL-10) were reduced in the diabetic (DG) group compared to the control and SAU-19 groups (Figure 7A–D and Figure 8A–D, *p* < 0.05). However, the *B. toyonensis* strain SAU-19 reduced the expression levels of proinflammatory cytokines and increased the expression of anti-inflammatory cytokines compared to the DG group (*p* < 0.05). There was no significant difference between the *B. toyonensis* strain SAU-19 and control (C) (*p* > 0.05) in the mRNA expression of all cytokines and the protein expression of IL-1β, TNF-α, and IL-10; however, the protein levels (ELISA) of IL-4 in the SAU-19 group were significantly lower than the control.

### 3.8. Effects of B. toyonensis Strain SAU-19 on Relative mRNA Expression of Genes Related to Glucose and Glycogen Synthesis in the Liver Tissues of HFD/STZ-Induced T2DM Mice

**Figure 9:**
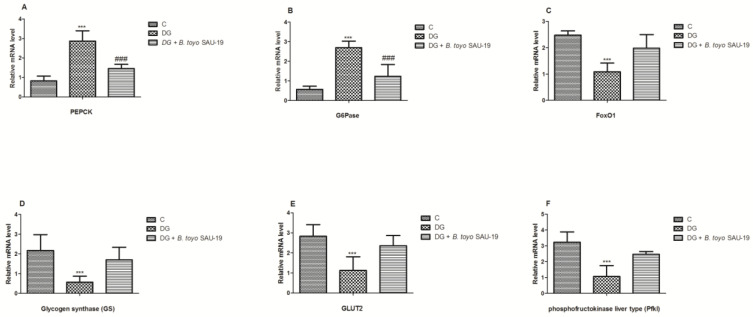
Effects of *Bacillus toyonensis* SAU-19 on the relative mRNA expression of glucose and glycogen synthesis-related genes in HFD/STZ-induced T2DM mice. (**A**,**B**) mRNA expression levels of genes related to glucose synthesis. (**C**–**F**) mRNA expression of genes related to glycogen synthesis. Values are shown as mean ± Sd. *** *p* < 0.05, DG compared with the control (C) and *B. toyo* SAU-19 groups. ^###^ *p* < 0.05, *B. toyo* SAU-19 compared with the control (C) group (*n* = 6).

As shown in Figure 9, the mRNA expression levels of genes related to glucose synthesis phosphoenolpyruvate carboxykinase (PEPCK) and glucose 6-phosphatase (G6Pase) were elevated in the T2DM mice group whereas Forkhead Box O1 (FOXO1), Glucose Transporter 2 (GLUT2), Glycogen synthase (GS), and phosphofructokinase liver type (Pfkl) were significantly reduced in the diabetic group compared to the normal mice group, but the administration of the *B. toyonensis* strain SAU-19 reverted this effect by downregulating the expression levels of PEPCK and G6Pase and upregulating the expression of FOXO1, GLUT2, GS, and Pfkl (Figure 9A–F, *p* < 0.05) compared to the diabetes mice.

## 4. Discussion

This study demonstrates the antidiabetic effect of *B. toyonensis* SAU-19 on mice with T2DM induced by HFD/STZ. We successfully modeled a T2DM mice model as basic characteristics of T2DM, such as weight loss, increased food and water consumption, and increase blood glucose, were evident in our study. Interestingly, the administration of *B. toyonensis* SAU-19 significantly improved hyperglycemia, insulin resistance, oxidative stress, and dyslipidemia. A study by Li et al. [19] reported that the body weights of HFD/STZ-induced diabetic mice were significantly lower than the control mice after the type 2 diabetes model was established. Similarly, in our study, we observed a decrease in the body weights of HFD/STZ-induced diabetic mice after the type 2 diabetes model was established. However, the feeding of *B. toyonensis* SAU-19 maintained the body weights compared to the type 2 diabetic mice. Kantas et al. [20] reported that *Bacillus toyonensis* could improve health and growth performance. Furthermore, we observed a reduction in the weights of the liver, kidney, and spleen in the diabetic group compared to the other treatment groups. This observation was consistent with the study by Zafar and Naeem-Ul-Hassan Naqvi [21], who reported a reduction in the body and organ weights of STZ-induced diabetes rats.

The immune organ weight and index are associated with immunity [22]. A study by Iftikhar et al. [23] reported that the weights of immune organs correlate with immune improvement; therefore, an improved immune organ weight signifies immunity improvement, whereas the opposite designates immunosuppression. From the results of our current study, we observed that the administration of *Bacillus toyonensis* SAU-19 increased the splenic weight and splenic organ index. Therefore, we concluded that *Bacillus toyonensis* SAU-19 stimulated the development of the spleen, hence enhancing immune performance in mice.

Recently, numerous probiotic strains have shown glucose-alleviating potential [24,25]. In our current study, *B. toyonensis* SAU-19 showed an effective antiglycemia activity via reduction and regulation of the glucose levels similarly to that in control mice throughout the experiment.

The liver is an important organ responsible for regulating glucose metabolism via assimilating excess blood glucose into glycogen [26]. The histopathological results obtained in this study revealed that *Bacillus toyonensis* SAU-19 did not cause any severe pathological changes in the liver tissues as compared to the diabetes group. Complications, such as hepatocyte structure disorder with extensive degeneration, congestion, and necrosis and inflammation, were observed in the liver of mice in the diabetes group. This was consistent with previous reports by Zeng et al. [1], who reported that probiotics *Lactobacillus paracasei* NL41 could prevent the pathological damage in the liver caused in HFD/STZ-induced mice.

Aspartate Aminotransferase (AST) and Alanine Aminotransferase (ALT) are markers of active liver inflammation and tissue damage [27]. Numerous studies have reported that diabetes causes a rise in the AST and ALT levels [28,29]. Similarly, in this study, we observed an increase in the levels of liver injury markers in the blood; however, the administration of *B. toyonensis* SAU-19 reduced the levels of these liver inflammation and damage markers. This result was consistent with the study by Mirmiranpour et al. [30], who reported that *Lactobacillus acidophilus* (probiotic) could reduce the levels of AST and ALT in type 2 diabetes patients. Furthermore, we also observed that liver glycogen levels were much higher in the *B. toyo* SAU-19 group, indicating that *B. toyonensis* SAU-19 reduced blood glucose by helping in the regulation and transport of high glucose loads from the blood to the liver to be converted into glycogen. This result was consistent with previous studies showing that *Saccharomyces boulardii* Tht 500101 significantly increased the storage of hepatic glycogen [31].

Insulin resistance is a pathophysiological disorder, which arises as a result of decreased insulin sensitivity in peripheral tissues [32]. In the present study, we observed that the glucose tolerance was unimpaired, and the increased levels of HOMA-IR were nullified in HFD/STZ-T2DM mice administrated *B. toyonensis* SAU-19, indicating that the administration of SAU-19 prevented or delayed the onset of T2DM by improving insulin resistance. This was consistent with the study by Balakumar et al. [33], who reported that the native probiotic strains MTCC 5690 and MTCC 5689 improve insulin resistance and type 2 diabetes.

Oxidative stress plays a crucial function in the onset of insulin resistance and T2DM [34]. Reactive oxygen species (ROS) activated by hyperglycemia and dyslipidemia induce injury in the liver [35,36]. Several probiotics have showed effective antioxidants activities [37]. In this study, supplementation of *B. toyonensis* SAU-19 significantly increased the activities of SOD and GSH but decreased the MDA activity. These results suggest that SAU-19 protected the body against oxidative damage, thus improving insulin resistance and reducing the injury to organs, such as the pancreas, liver, and kidney.

Systemic and subclinical inflammation is associated with type 2 diabetes mellitus [38,39]. The inflammatory process is characterized by increased levels of inflammatory factors, such as C-reactive protein (CRP) or high-sensitivity CRP (hs-CRP) and inflammatory cytokines [40]. In the hepatocytes, the process of inflammation causes the production of numerous acute-phase proteins, such as ferritin, which enhances insulin resistance [39]. The results from this study showed that the expression of proinflammatory cytokines (IL-1β and TNF-α) in the liver was elevated while the levels of anti-inflammatory cytokines (IL-4 and IL-10) were reduced in the diabetic group compared to the normal group; however, the administration of *B. toyonensis* SAU-19 reverted these effects. This result is consistent with the study by Liu et al. [41], who reported that *Lactobacillus rhamnosus* GG culture supernatant (LGGs) could reduce liver inflammation and injury in a high-fat/high-fructose diet and intermittent hypoxia exposure-induced metabolic dysfunction.

Gluconeogenesis and glycogenolysis are two major pathways for endogenous glucose production [42]. PEPCK and G6pase are two key enzymes of hepatic gluconeogenesis [43]. PEPCK and G6pase catalyzes the process of gluconeogenesis in the liver and thus is associated with glucose production [6]. Increased expression of PEPCK and G6pase in the liver has been linked with the onset of type 2 diabetes [44]. FOXO1 is a member of the forkhead family transcription factors, which directly binds to PEPCK and G6pase target DNA sequence to control their expression in the liver [45]. Studies have proved that the inhibition of FoxO1 decreases hepatic gluconeogenesis and improves glucose metabolism in animals with T2DM [46]. GS is the rate-limiting step for glycogen synthesis, and activation of GS by decreasing its phosphorylation results in increased glycogen synthesis [47]. GLUT2 is a bidirectional glucose transporter and a transmembrane carrier protein mostly found in the liver, kidney, and pancreas and is involved in supporting the passive movement of hexoses through the cell membranes [48]. The high expression levels of hepatic GLUT2 mRNA reported in this study were similar to those reported by Matsuzaka et al. [49] and Narasimhan et al. [50] in rats, Jung et al. [51] in mice, and Okamoto et al. [52] in the HepG2 cell line. The upregulation of GLUT2 expression in diabetes mice may increase the hepatic glucose output since it was suggested that GLUT2 transports glucose from the liver when the intracellular concentration of glucose exceeds its concentration in the plasma [53,54]. In cellular respiration, phosphofructokinase-1 (PFK-1) controls the oxidation of glucose [55]. An increase in the levels of PFK-1 has been reported to increase glucose metabolism [42,56]. The results from this study showed that the mRNA expression levels of PEPCK and G6pase genes were higher in the diabetic group as compared to the control group; however, the administration of *B. toyonensis* SAU-19 reduced the expression of these genes, indicating that *B. toyonensis* SAU-19 suppresses hepatic gluconeogenesis. This result was consistent with the study by Yadav et al. [57], who reported that *Lactobacillus rhamnosus* MTCC: 5957, *Lactobacillus rhamnosus* MTCC: 5897, and *Lactobacillus fermentum* MTCC: 5898 reduced the mRNA expression of PEPCK and g6pase. Furthermore, the expression of FOXO1, GS, GLUT2, and PFK-1 in the treatment group was elevated compared to the diabetes group. This indicated that *B. toyonensis* SAU-19 reduced T2DM by upregulating genes related to glycogen synthesis and excess glucose transport in the liver. This result was similar to the study by Kim et al. [58], who reported that *Bifidobacterium lactis* HY8101 upregulated the glycogen synthesis-related gene pp-1 and GLUT4 and downregulated the hepatic gluconeogenesis-regulated genes (PCK1 and G6PC) in diabetic mice.

This study reported on the antidiabetic activity of *Bacillus toyonensis* SAU-19; however, the molecular mechanisms involved in the treatment of T2DM were not fully reported. Therefore, we suggest that further studies should be conducted to elucidate the potential molecular mechanisms that the *B. toyonensis* strain SAU-19 uses to prevent or treat type 2 diabetes.

## 5. Conclusions

In conclusion, this study demonstrated that the *B. toyonensis* strain SAU-19 has an excellent antidiabetic effect in HFD/STZ-induced T2DM mice. The potential mechanism of this effect might be related to decreasing insulin resistance and oxidative stress, upregulating genes related to glycogen synthesis and glucose transport, and improving lipid profiles. Therefore, from the results, *B. toyonensis* SAU-19 could be used for the treatment of T2DM. However, further studies are still needed to clarify the detailed mechanisms of action by validating the efficacy of *B. toyonensis* SAU-19 through human clinical trials.

## Figures and Tables

**Table 1:** Feed composition.

Normal Diet		High-Fat Diet	
Ingredients	Content g/kg	Ingredients	Content g/kg
Water	94	Water	93
Protein	190	Protein	134
Fat	51	Fat	143
Fiber	36	Fiber	27
Ash	62	Ash	44
Calcium	11.3	Calcium	8.3
Phosphorus	8.6	Phosphorus	7.1

**Table 2:** Primers used for the real-time PCR analysis.

Gene Name	Primer	Sequence (5′ and 3′)	Product Length (bp)	Annealing Temperature (°C)	Sequence Number
IL-1β	Forward	TGAAATGCCACCTTTGACAGTG	141	60.18	NM_008361.4
	Reverse	ATGTGCTGCTGCGAGATTTG			
PEPCK	Forward	GACAGACTCGCCCTATGTGG	98	59.90	NM_011044.3
	Reverse	GGCACTTGATGAACTCCCCA			
IL-4	Forward	GTACCAGGAGCCATATCCACG	130	60.18	NM_021283.2
	Reverse	TTCGTTGCTGTGAGGACGTT			
IL-10	Forward	GGGGCGAGTGTAACAAGACC	109	60.27	XM_036162094.1
	Reverse	GCAGAGGAGGTCACACCATTT			
TNF-α	Forward	CCCTCACACTCACAAACCAC	211	59.82	NM_001278601.1
	Reverse	ATAGCAAATCGGCTGACGGT			
g6pc	Forward	GTTTGGTTTCGCGCTTGGAT	95	59.82	NM_008061.4
	Reverse	GCCGCTCACACCATCTCTTA			
FoxO1	Forward	AGTGGATGGTGAAGAGCGTG	96	60.04	NM_019739.3
	Reverse	GAAGGGACAGATTGTGGCGA			
GS	Forward	AGGATGAATTCGACCCCGAG	81	55.00	NM_030678.3
	Reverse	CAGTGTAGATGCCACCCACC			
Glut2	Forward	GATCACCGGAACCTTGGCTT	76	55.00	NM_031197.2
	Reverse	CACACCGATGTCATAGCCGA			
Pfkl	Forward	AAAGCGGCGTGTGTTCATTG	73	60.39	NM_008826.5
	Reverse	AGCAATGCCGGTCACAGTAG			
β-actin	Forward	TTCGCGGGCGACGAT	297	58.57	NM_0077393.5
	Reverse	CATCTTTTCACGGTTGGCCT			

## Data Availability

The data presented in this study are available on request from the corresponding author.
